# Supplementary material for: Modelling Exposure by Spraying Activities—Status and Future Needs
Source: Int J Environ Res Public Health. 2021 Jul 21;18(15):7737. doi: 10.3390/ijerph18157737 (PMC8345348; doi:10.3390/ijerph18157737)
Supplement: Supplementary file 1 [file ijerph-18-07737-s001.zip › PRISMA_2020_flow_diagram_20210707.pdf]

Modelling Exposure by Spraying Activities - Status and Future Needs

Supplementary information: Documentation of the systematic literature search

The systematic literature search was performed before and after the symposium at the ISES-ISIAQ Conference 2019. The search was verified in June 2021.

1. **Search in Databases:**

In Pubmed, WebOfScience, Scopus:
Search term 1: exposure AND spraying AND model AND (occupational OR work* OR consumer)

In Pubmed:
Search term 2: exposure AND (model OR tool) AND (robustness OR validation)

The resulting papers are the records from the databases after identification. Duplicates were removed, and the remaining records separately screened and evaluated.

To select potentially relevant records, in a first step an automatic screening of the records were performed using Endnote. Both datasets were limited separately on specific search terms in the Title or in the Keywords:

- Title: evaluation AND model
- Title: spray OR reliability OR REACH
- Keywords: spray OR reliability OR REACH OR validation

The resulting records are merged into one dataset, containing the potentially relevant papers for retrieval.

In a second step the remaining paper (n=308) were further screened manually in Title and Abstract. By human expert, the relevant paper for the review for modelling exposure during spraying activities were selected. For example all records about effects and validation of chemical analytical methods for monitoring purposes were ignored.

2. **Search on Websites of Organizations, including search for secondary citation and expert knowledge:**

Alongside the literature search via databases and registers, we also conducted a search via other methods:

- Search via organisation websites such of ECHA, EFSA, EPA, OECD and WHO:

We searched high level organisation websites such as ECHA, EFSA, EPA, OECD and WHO. In order to narrow down the search, we limited it to certain sub-pages or specific search words, which are described in more detail in the following. On the ECHA website a focus was laid on the REACH guidance on information requirements and chemical safety assessment, the use maps library and the BPR Volume III Human health guidance on in-formation requirements and exposure assessment and evaluation. The search on the EPA website focused on information related to the EPA ExpoBox. The OECD website search focused on the OECD activities on exposure assessment and the emission scenario documents. The WHO website was searched as whole. The keywords “exposure model” or “spray” were searched for in the documents on these sub webpages. The EFSA website was searched for the search words “exposure”, “model”, “consumer” and “operator”. This search resulted in a high number of hits. However, also the key publication EFSA, 2014 was identified. Based on this publication, the search string was altered to “exposure calculator”.

- Collection of spray models and subsequent search on model and project websites:

We have compiled a list of the models known to us for estimating the exposure of workers and consumers from spraying activities. This list was updated after the literature search via databases and registers. We excluded obsolete models that are no longer available and are no longer used in the European chemical regulatory processes from further search. In addition, models were sorted out that are in a very early development or demonstration status with one exception the dART model. The list served as a starting point to search the model and project

websites for additional peer-reviewed references and grey literature such as model documentation and background documents.

- Secondary citation search via key publications

Especially for the evaluation of the performance of the models when applied to spray activities four key publications (Spinazze et al 2019, Lee et al 2019, Delmaar and Meesters 2020) were identified based on the literature search via databases and registers. On the basis of this key publications a secondary citation search was conducted whereby the citations in the supplementary material of the papers were also taken into account.

- Collection of references via our expert knowledge

Last but not least, we also considered references that were not identified by any of the search strategies described above, but were known to one of the authors through expert knowledge.

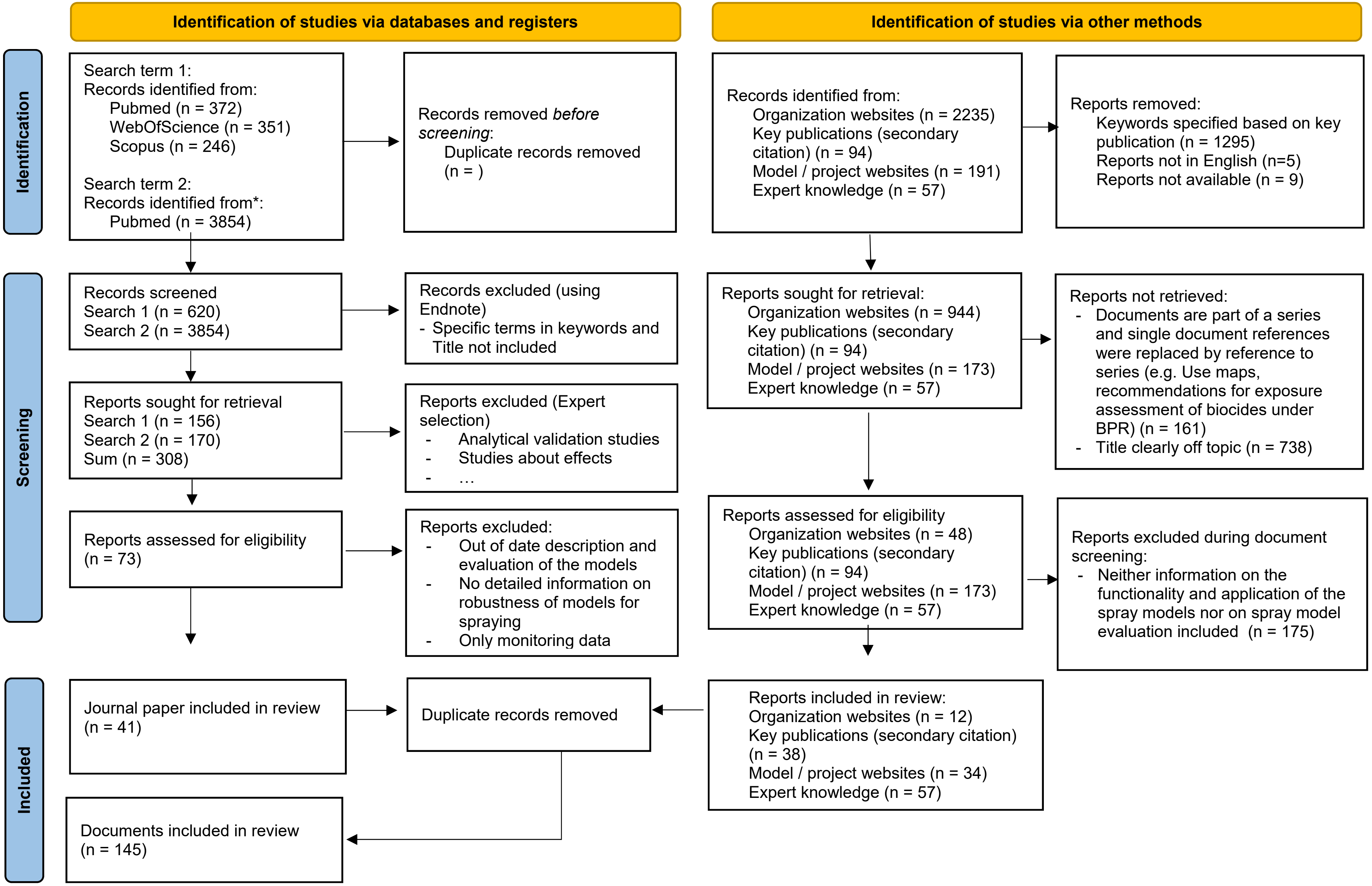

Figure S1: **PRISMA 2020 flow diagram for new systematic reviews which included searches of databases, registers and other source**
